# Recognising and Treatment Seeking for Acute Bacterial Meningitis in Adults and Children in Resource-Poor Settings: A Qualitative Study

**DOI:** 10.1371/journal.pone.0068163

**Published:** 2013-07-04

**Authors:** Nicola A. Desmond, Deborah Nyirenda, Queen Dube, MacPherson Mallewa, Elizabeth Molyneux, David G. Lalloo, Robert S. Heyderman

**Affiliations:** 1 Malawi-Liverpool-Wellcome Trust Research Programme, Blantyre, Malawi; 2 Liverpool School of Tropical Medicine, Liverpool, United Kingdom; 3 Queen Elizabeth Central Hospital and College of Medicine, University of Malawi, Blantyre, Malawi; Oxford University, Viet Nam

## Abstract

**Objective:**

High mortality burden from Acute Bacterial Meningitis (ABM) in resource-poor settings has been frequently blamed on delays in treatment seeking. We explored treatment-seeking pathways from household to primary health care and referral for ABM in Malawi.

**Design:**

A cross-sectional qualitative study using narrative in-depth interviews, semi-structured interviews and focus group discussions.

**Participants:**

Adults and children with proven and probable acute bacterial meningitis and/or their carers; adults from urban and peri-urban communities; and primary health care workers (HCW).

**Setting:**

Queen Elizabeth Central Hospital (QECH), urban and peri-urban private and government primary health centres and communities in Blantyre District, Malawi.

**Results:**

Whilst communities associated meningitis with a stiff neck, in practice responses focused on ability to recognise severe illness. Misdiagnosis of meningitis as malaria was common. Subsequent action by families depended on the extent to which normal social life was disrupted by the illness and depended on the age and social position of the sufferer. Seizures and convulsions were considered severe symptoms but were often thought to be malaria. Presumptive malaria treatment at home often delayed formal treatment seeking. Further delays in treatment seeking were caused by economic barriers and perceptions of inefficient or inadequate primary health services.

**Conclusions:**

Given the difficulties in diagnosis of meningitis where malaria is common, any intervention for ABM at primary level must focus on recognising severe illness, and encouraging action at the household, community and primary health levels. Overcoming barriers to recognition and social constraints at community level require broad community-based strategies and may provide a route to addressing poor clinical outcomes.

## Introduction

More than one million cases of acute bacterial meningitis (ABM) amongst adults and children occur annually in sub-Saharan Africa (SSA) [1], associated with high burden of death and disability [2]–[4]. In settings such as Malawi (human development ranking of 171/187 countries [5]), 41% of children and up to 50% of adults hospitalised with ABM die [6]. Additional deaths are likely to occur at home without medical intervention, particularly amongst young children [1].

Since length of time between onset of symptoms and development of life-threatening illness in ABM is usually short, early recognition and prompt treatment are vital for effective management. In Malawi, it is estimated that 37% of illness episodes are managed at home without external advice and an additional 16% seek help from traditional healers [7]. It has been shown that although 72% of children receive medication at home for febrile illness, only 12% receive appropriate treatment [8]. The context for health seeking behaviour for ABM in Malawi and other resource-poor countries is therefore complex, involving a balance between structural issues such as costs and accessibility [9], social concerns [10], assessments of illness severity [11] and perceptions of service provision [12]. Families living in severe poverty frequently have to weigh up the needs of the sick against provision for the rest of the family.

Whilst ABM has been the focus of much clinical research in resource-poor contexts such as Malawi [1], [2], [6], [13], and there have been a number of qualitative studies on treatment seeking [8], [14]–[16] there have been no studies exploring complex community level responses and social constraints in response to ABM in resource-poor settings. We have conducted a qualitative study to explore the decisions people make and pathways followed when faced with the symptoms of ABM within the household, community and at different stages of the health seeking process.

## Methods

### Ethics Statement

The study was approved by the Liverpool School of Tropical Medicine (LSTM), Research Ethics Committee in the UK, and the University of Malawi College of Medicine, Research Ethics Committee (COMREC) in Malawi (approval number: P10/09/832).

All participants gave written informed consent (or a witnessed thumb print if illiterate) to take part in the study and to use anonymised data for publication.

### Design and Setting

We conducted a cross-sectional qualitative study in the district of Blantyre, Malawi, recruiting from three groups: since the majority of those defined as having probable meningitis have the same outcome as those defined as having proven disease [2] we purposively selected patients with both proven and probable [6], [13] ABM (adults and children) and/or their carers at Queen Elizabeth Central Hospital (QECH) (a large district and referral hospital); adults from urban and peri-urban communities; and primary health workers (HCW) from urban private and government health centres. We defined proven ABM according to standard criteria established for SSA contexts and applied at QECH. For clinical care decision-making probable meningitis in paediatrics is considered meningitis, even if culture negative. For adults, a proven case was defined with one of the following parameters: the presence of culture positivity in the cerebrospinal fluid (CSF) of a bacteria known to cause meningitis, a positive latex agglutination test on the CSF, or an organism known to cause bacterial meningitis seen on gram-stained CSF in the presence of an appropriate clinical syndrome. A probable case of ABM was defined as clinical evidence of ABM with a CSF white cell count of >100 cells/mm^3^ in HIV negative and >5 cells/mm^3^ in HIV positive individuals with at least presence of 50% neutrophils in culture negative CSF.

### Recruitment and Sampling

For the ABM group, we recruited participants (patient/carer pairs) for in-depth narrative interviews identified as adult and paediatric hospital in-patients between March and November 2010 using a purposive sampling framework based on patient outcomes since we expected differences in treatment seeking behaviours across adults and children [17], [18] and across outcomes [19]: adult with positive outcome (recovery with no major disability); child with positive outcome; adult with negative outcome (death or long-term disability); and child with negative outcome. Recruitment was a two-staged process beginning with initial informed consent from carers of patients admitted to hospital with proven or probable bacterial meningitis. Those providing initial consent in hospital were followed up and a second recruitment stage with full written consent took place during a home visit. Interviews were conducted immediately or during a follow up visit and included the patient alone, the patient and carer or carer alone. Parent carers were interviewed in the presence of resident children.

For the community group, we recruited 96 members into 8 focus group discussions (FGDs) from established community-based social groups such as women’s or microfinance groups, identified and approached with assistance from community leaders within one urban and one peri-urban ward in Blantyre, Malawi.

For the HCW group we conducted semi-structured interviews (SSI) with 20 private and government primary and community health service workers stratified by cadre proportional to the number of staff in the same wards.

### Interviews

Interviews with patients and carers were conducted by an experienced female, Malawian Social Scientist who had not worked in the hospital previously (DN). These began with an open question asking the participant to describe the experience of meningitis and provide a treatment seeking narrative. This was followed by a guided set of topics to identify critical incidents [20] during treatment seeking trajectories, explore issues in depth and situate these within the broader context of individual lives. Guided topics included home life, social influences on treatment seeking such as social relationships, decision-making and social capital and general treatment seeking practices. FGDs conducted by DN explored lay understandings of meningitis within broader perceptions of health and illness, particularly how experiences with primary health services impact on decisions around treatment seeking. Interpretations of particular symptoms and combinations of symptoms were explored using a series of simple pictorial images developed with local artists based on the Meningitis Research Foundation, UK (MRF) symptom cards (www.meningitis.org). Interviews with HCW were conducted by ND, a senior researcher from the UK, resident in SSA for 12 years. Topic guides for SSI with primary health providers were developed from preliminary findings and focused on interactions between providers and patients, techniques used to recognise and respond to severe illness and barriers to action as perceived by health personnel.

Patient/carer interviews lasted on average 1 hour, SSIs on average 45 minutes, whilst FGDs took place over 2 and ½ hours and were broken up with refreshments. Interviews with patients and carers and FGDs were conducted in Chichewa, recorded, transcribed verbatim and translated into English. Interviews with HCW were conducted at primary health facilities in English, recorded and transcribed. All transcripts were checked for quality and accuracy by those who conducted the interviews.

### Analysis

Using thematic content analysis, we developed an initial coding framework based on a combination of research questions and a grounded theory approach to inductively document emerging themes from the data by independently coding 2 patient/carer interviews, 1 FGD and 1 SSI with health providers, triangulating through multiple and independent coding (ND & DN). The coding frameworks were compared, refined and updated during regular meetings as coding of transcripts progressed using NVIVO 9.0 (QSR International, Melbourne, Australia) for qualitative data analysis. We used this thematic coding to guide the development of a treatment-seeking framework to explore and compare individual cases for each theme using constant comparison approaches [21]. This enabled us to describe the critical incidents [20]that occurred on the pathway to care and link these to broader elements of individual, social contexts. For each of the main themes we developed data summaries to triangulate findings across data sources and balance abstract discussions of symptoms and illnesses reported during FGDs against lived experiences of patients, carers and health workers. Direct quotations from research participants cited were selected explicitly to represent dominant themes emerging from the thematic analysis.

## Results

### Respondents

We recruited 43 ABM patient/carer pairs during stage 1 and interviewed 17: five carers of children following a positive outcome; four carers of children following a negative outcome; four adults and carers following a positive outcome; and four carers of adults following a negative outcome. Reasons for non-participation included loss to follow-up in the community or participants having changed their minds. Attempts to follow up recruited pairs continued according to recruitment date until data saturation was reached [22]. The median age of adult ABM patients interviewed was 28 years (range 18 to 48). All children with ABM were under 5 years. (Table 1 shows patient demographics against outcome for proven and probable adult and paediatric cases.) We recruited an additional 96 community individuals and 20 HCW to contextualise patient/carer data.

**Table 1:** Summary of proven and probable ABM paediatric and adult cases interviewed by outcome and HIV status (where known).

	Age	Sex	HIV status	Outcome
**Confirmed ABM**				
Paediatric	0–5	female	n/k	Died
	0–5	female	n/k	Discharged with disabilities
	0–5	male	n/k	Discharged
	0–5	female	n/k	Discharged
	0–5	male	n/k	Discharged
	0–5	male	n/k	Discharged
Adult	31–40	male	positive	Died
	21–30	male	n/k	Died
	16–20	male	negative	Discharged
	21–30	female	positive	Discharged
	21–30	female	n/k	Discharged
	21–30	male	negative	Discharged
**Probable ABM**				
Paediatric	0–5	female	n/k	Discharged with disabilities
	0–5	female	n/k	Died
	0–5	female	n/k	Discharged
Adult	21–30	female	positive	Died
	31–40	male	n/k	Died
	31–40	male	n/k	Died

### Qualitative Findings

We identified several themes that ran through each of our patient, community and HCW groups related to recognition of meningitis and subsequent action.

### Recognition of Meningitis as Dangerous

HCWs claimed knowledge of the signs and symptoms of meningitis but also recognised difficulties in diagnosis. Misdiagnosis with malaria occurred in seven of nine and five of eight children and adult illness episodes respectively.

Recognition at household level focused on ability to identify when illness is severe enough to warrant treatment seeking rather than ability to recognise signs and symptoms of ABM. However, when discussions focused on meningitis explicitly, this was recognised as a dangerous disease, particularly associated with stiff neck.

*‘Maybe when you realise … it’s when a child’s body is stiff. When you are going to the hospital maybe you are late, it’s a dangerous disease’.* Women’s group participant, peri-urban ward

*‘The problem with meningitis is that it looks like a simple disease but it is dangerous … if you wait until the child’s body parts become stiff … it is very dangerous’.* Mother of female child who survived proven meningitis with disabilities

Patients and carers had not recognised the illness as meningitis until diagnosed in hospital in all but two cases; in these, prior experience led to earlier suspicion of meningitis following neck retraction.

### Recognition of Severe Illness

Community discussions centred on specific symptoms associated with ABM, particularly focusing on severe illness: fever, vomiting, severe headache, stiff neck, drowsiness/vacancy, confusion/delirium, rash and seizures/convulsions. Of these, all groups defined seizures/convulsions as most severe.

*‘Because this disease is very dangerous, if a person has convulsions it doesn’t take time, a person can die so it’s a dangerous disease’.* Women’s group participant, peri-urban ward

Seizures were often associated with malaria which delayed timely recognition and treatment seeking since malaria was often treated at home with purchased anti-malarials to avoid long waiting times in primary health centres. Seizures were also associated more directly with epilepsy as an innate, traditional illness caused by ‘internal worms’ which was described as requiring traditional treatment, and delayed onset of biomedical treatment seeking.

*‘Seizures or convulsions it’s a big problem. Because there’s this disease, epilepsy (matenda) that often attacks children, … worms may come in the person’s head and he may start having seizures or convulsions.’* Men’s group participant, peri-urban ward

Severe headache was not considered as real illness and home-based remedies were sought according to perceived cause: painkillers if biomedical & traditional medicine if indigenous illness.

*‘He would refuse [to go to hospital] “no, It’s only the head which is hurting, just buy me some medicine’.* Female widow of husband with probable meningitis

Severity was often assessed by the effectiveness of symptom control with popular medicines. Less severe illness could be treated with traditional medicine but more severe treated with biomedicine.

When patients and carers recounted illness narratives of meningitis, discussions featured general references to recognition of illness severity. References commonly related to the extent to which ‘normal’ social life was sufficiently disrupted by illness to warrant treatment seeking. In Table 2 we present issues around social disruption that impacted on recognition at community level.

**Table 2:** Recognition of severity of illness.

Recognition	Social group	Key quotes	Source
Lack of mobility	Adults and infants	‘And you may see a person looking very weak maybe he is laying down butno power/strength to get up, and he cannot walk on his own.’	FGD Male Youth, Peri-urban ward
		‘But that day, he could not stand up when he tried to, he could not sit down.So I knew that that day, he was seriously ill’.	Female widow of husband with probable meningitis
		‘So when he reached a point where he became very sick, he failed to sit down,he failed to stand up. It’s when he (neighbour) suggested “this person shouldnot stay here”. Yes, I can say the severity of the illness was what made us go’.	Female widow of husband who died of proven meningitis
Inability to work	Adult men	‘So from there he was still working but it reached a point that he couldn’twork like his strength was reducing … so when he started being weak likethat it was when he changed from there it was when he was picked up by otherpeople who work at Queens’	Father of young man who died of proven meningitis
Inability to performusual domestic role	Adult women	‘What concerned me was that the woman is sick, *but also what the woman* *was supposed to do at home*. I was going to miss it all, I should rush to thehospital that this problem should not grow’	Husband of woman who survived proven meningitis
Refusal to eat	Adults and infants	‘: What worried you about this disease? R: Because she didn’t eat anything,she refused’	Mother of infant survivor of proven meningitis
		‘She was not receiving the food that it is why we noticed that she was seriouslyill, let’s take her to the hospital’	Husband of female survivor of proven meningitis
		‘I: Did you ever think that the child was seriously ill? R: Yes I: How? R: Sometimeshe would refuse to suck milk from the breast, just crying’	Mother of infant survivor of proven meningitis

### Delays in Treatment Seeking Following Recognition of Severe Illness

We identified social and economic barriers to initiating appropriate and prompt action following recognition of severe illness within the household, as well as perceptions of poor service response based on previous experiences with healthcare professionals.

### Need to Validate Severity

At the household level recognition of illness severity was not sufficient in itself for the patient or carer to initiate treatment seeking. This was rarely initiated without some form of collective decision-making and social validation either from senior household members or peers within the broader community. The type of validation depended on a combination of the gender and social position of the carer and patient (Table 3). Where men were the principle carer or decision-maker within the household they made decisions alone, in part due to their increased access to financial resources but also due to the respective power positions between husband and wife.

**Table 3:** Delays in action: need to validate severity and social position.

Patient	Carer	No. of cases	Type of validation before treatment seeking
Adult male	Adult female	2	Wives seek confirmation of severity by husband himself, adult relatives or neighbours. *‘my brother-in-law said “you must force him” but I explained that he wouldn’t listen to me so he told him that he should go to hospital. so he my husband said “I have heard, I will go”’*
		3	Male patients make decision alone whether to seek treatment. *‘Who decided he should go?’ ‘He decided himself’*
Adult female	Adult male	1	Husband makes decision with extended family in matriarchal society *‘That was decided by my brother-in-law … what should we do? “Lets take this person to the hospital so it was like we just shared the same idea with him”*
		1	Husband makes decision alone as socially alienated in wife’s village *‘some relatives were saying that we should go to the traditional healer but I said, fine, even if they call me abnormal or they say I am making a decision without consulting relatives, I will give my opinion whether they like it or not, but I will go to the hospital’*
Infant	Adult female	8	Mothers seek confirmation of extended family and friends more often than husbands or fathers *‘It was the same friends who told me that a child’s illness appears to be small while the disease is big because the child doesn't explain … it is better that you go to the hospital’*
Infant	Adult male	1	Father made decision alone since fails to trust neighbours in wife’s village *‘Since I arrived I have never seen anyone coming here to greet me, these people didn’t welcome me here so how can I be free? I don’t feel comfortable. That is how we live here. With this disease … they just see me going to the hospital with her and then coming back’*
Adult female	Adolescent daughter	1	Daughter sought confirmation from neighbours realising that confirmation from father/husband would delay treatment which saved the mother‘*so when the child saw that things were getting worse its when she phoned my friends and four of them came’*

### Household Economics

Seeking treatment following recognition of severe illness was often constrained by poverty and lack of funds for transport.

*‘It affected us, it was in the middle of the month. We saw that we had nothing, what were we going to do? So I ran and borrowed here and there ….’* Wife of male adult who survived proven meningitis

Reliance on others to fund emergency treatment reinforced the need for social validation of illness severity. Delays could result if illness was not recognised as severe enough to warrant expenditure.

*‘Since I didn’t have any money, I waited for my father to come in the evening. He granted my request and told me he would give me the money the following morning.’* Single mother of child who died of proven meningitis

In households where the man was the main income–earner, he was also largely in charge of household expenditure and was often reticent to spend time and money on illness unless it was severe. This often delayed treatment seeking for adults (see Table 3).

In contrast, women as the main carers often managed child illness, and decisions were regularly made without consultation with the father and treatment initiated once resources were obtained. Success in accessing resources was dependent on social validation (see Table 3) in addition to availability of resources within the household and social networks. The consequences of this complex treatment seeking process and reliance on economic resources are well recognised in the community.

*‘People were saying “that’s the funeral” it was just like the child is sleeping. People asked the family “why did you not go to hospital?” They said, “they [HCW] told us to go to Queens [regional referral hospital] but we didn’t have transport [money]. The wife delayed because of borrowing money and the child died on the way.’* Female participant in FGD

### Perceptions of Service Quality

Decisions to seek conventional medical treatment during early stages of illness were clearly affected by perceptions of the quality of service provided through the primary health system. Reports amongst primary health workers and patients highlighted a lack of quality care with long waiting times, presumptive diagnosis without examination, verbal mistreatment and erratic drug availability.

*‘We health workers, we usually shout at them “you are not supposed to tell me what the child is suffering from. You are supposed to tell me the signs and symptoms”’* HCW from urban Blantyre

*‘Before you finish talking about the disease you are suffering from, you find that they have already written in the book and thrown it to you.’* Male participant in FGD

Patients were frequently diagnosed initially with malaria and prescribed anti-malarial medication. In most cases patients were not told to return if symptoms persisted, often prompting recourse to alternative service providers. HCW interviews revealed that it is not uncommon for patients to be blamed for persistent illness, especially mothers who returned with sick children, on the assumption that they had failed to give the child the prescribed medication.

*‘The woman comes, you think that it is not malaria, you give them painkillers, they go home. Then after 2/3 days they come back, they say there is no improvement. We think that woman is demanding for nothing. We send them home, but we are supposed to investigate further’* HCW in urban ward

Women were often habituated to these conditions through regular attendance at under-5 clinics and therefore sought treatment for infants promptly at government services. However men in particular and adults in general often chose to remain at home, self-treating early stage symptoms as malaria, until symptoms were severe enough to circumvent primary clinics and seek emergency care at hospital.

## Discussion

We have shown the complex social environment within which people recognise and respond to severe illness in a SSA setting, providing insights into the broader context of health seeking behaviour. Our findings reflect studies elsewhere that highlight the importance of gender and social position in treatment seeking decisions made within the complex dynamics of households and communities [8], [9], [15]–[17], [23]–[26]. Research in Nepal has shown that gender is relevant for childhood illness in perceptions of illness but not in treatment seeking [25] whilst data from Kenya and Ghana has highlighted the link between gender and decision-making for seeking treatment [17], [26]. Here we have shown that gender of the carer impacts on the need to socially validate illness prior to treatment seeking, and that men made decisions alone whilst women rarely did so. This is attributable to increased access to financial resources amongst men but also to respective decision-making powers and breakdown positions [23] of men and women within the household. We have also shown that the recognition of illness as severe enough to warrant treatment, the views of key family members, the likely disruption to everyday life and associated costs all influence health-seeking behaviour.

Responses to ill health in families and communities are informed by what has been termed a lay epidemiology of illness [11], [27], described as ‘*a scheme in which individuals interpret health risks through the routine observation and discussion of cases of illness and death in personal networks and the public arena, as well as from formal and informal evidence arising from other sources*’ [11]. This lay epidemiology focuses in this case on the interpretation of disease severity and the resulting timeliness of appropriate treatment seeking through assessments of social disruption, as interpreted by household and social networks. We found that even in the case of life-threatening illness, the view of social networks often supersedes individual choice for treatment seeking [28]. Table 3 shows how the requirement for validation by others is influenced by the socio-demographic profile of the patient and carer. We have also shown that social validation is necessary to ensure financial assistance for unforeseen health expenses and that access to economic resources often reflects gendered power relations. These costs to the family all too frequently remain a prohibitive burden despite universal provision of free health care in Malawi.

We found that real or perceived inadequacies in primary health care impact on decision making and treatment response to severe illness [14] without often a clear understanding of the factors underlying these inadequacies such as overburdened clinics. This lay response to service quality needs to be considered when designing primary health services. Whilst, for example, a pragmatic strategy of presumptive diagnosis without examination or full consultation makes sense from a public health, high workload perspective, it may have unforeseen consequences on the timeliness of presentation for care. Malaria provides a well-documented example of delays in seeking treatment due to expectations of presumptive treatment and broader issues concerning quality of service at primary health level. It is estimated that 70% of malaria episodes are dealt with therapeutically within the household and malaria over-diagnosis at primary health level is between 30 and 70% [14]. Presumptive diagnosis of malaria, often associated with seizures, frequently caused delays in seeking appropriate care amongst our cases of ABM at both household and health centre level. There is substantial evidence of the difficulties of differentiating febrile illnesses in SSA settings including Malawi [29]. We have shown how such difficulties at primary health level exacerbate the difficulties in diagnosing ABM, experienced in less tropical settings [30]. We have also shown how seizures, when associated with epilepsy were considered an innate, traditional illness, thought to require traditional treatment. Our findings associating epilepsy aetiology with ‘internal worms’ reflect research elsewhere in several Eastern and Southern African ethnic groups as a common, indigenous theory of ill health [31]–[33]. This ascription of cause was thought to require traditional treatment and often delayed onset of biomedical treatment seeking.

We based symptom recognition on cards developed by the Meningitis Research Foundation for advocacy purposes in the UK. Whilst haemorrhagic rash is rarely observed in Africa, all other symptoms related to meningitis present similarly across contexts. Furthermore, although there are clearly cultural differences, there are some striking similarities between our study in Malawi and research conducted in resource-rich settings [34], [35]. These include difficulties in recognition of ABM or indeed severe illness by both families and HCWs, barriers to definitive referral at primary health level and insufficient emphasis on parental consultation [34].

### Limitations and Strengths of the Study

Patient/carer interviews were conducted generally at least two weeks after hospital discharge or death, and in some cases additional delays were experienced. This may have affected precise recall of acute illness events and treatment seeking details. Equally rationalisation of events and the desire to avoid blame may have altered the narratives given. We were not able to discern an association between outcome and number of days before initiating treatment due to a qualitative emphasis on understanding behavioural influences.

All our patients were recruited from QECH, a tertiary, regional, referral hospital which also acts as a district hospital for Blantyre District, providing comparatively high levels of care and treatment. However, patient profiles and pathways to QECH reflect those elsewhere in Malawi since these pass through the primary health system. Similarly, participants in FGDs were recruited from specific social groups so FGD participants may have had greater access to social capital than non-group members. However triangulation of findings between individual interviews and FGDS and restriction within FGDs to understanding perceptions of illness and causation theories helped to maximise generalizability.

Whilst we present one specific geographical and disease context, similarities across countries in social life, poverty and health service constraints make the findings relevant, transferable and salient to understanding the complex nature of treatment seeking for severe febrile illness in many sub-Saharan African settings.

### Conclusions and Implications

This study has explored treatment-seeking pathways in Malawi for acute bacterial meningitis to identify reasons for late presentation, identified as a major contributor to increased mortality. Diagnosis of ABM as a distinct and distinguishable condition is fraught with difficulties at both community and primary levels. We suggest that any intervention for ABM should focus on recognition of and response to severe illness and should not depend upon the need to reach a definitive diagnosis before urgent referral to more advanced tertiary care. Recognition may be improved through adaptation of Emergency Triage, Assessment and Treatment (ETAT) practices for primary care services currently aimed at tertiary care admissions departments in resource-poor contexts [36], [37].

Broader community-based strategies for health promotion to address recognition and social constraints, such as decision-making norms, to immediate action in response to severe illness may provide an additional route to addressing poor outcomes associated with severe febrile illness and ABM in particular [38].
